# Turning the tide towards cardiovascular prevention

**DOI:** 10.1186/s12916-022-02618-4

**Published:** 2022-11-09

**Authors:** BMC Medicine

**Affiliations:** The Campus, 4 Crinan Street, London, UK

Cardiovascular disease (CVD) can begin early and progress over one’s lifetime. Chronic disease limits the ability of therapies to reverse pathophysiology once symptoms present. An estimated 17.9 million lives are lost annually to CVD, making it the leading cause of death globally. Current treatments are mostly reactive and used primarily when a patient becomes ill. So, how are the medical and public health communities tackling CVD, and what will disease prevention look like in the coming decades?

Recent results from the highly anticipated DELIVER trial exemplify the limitations of contemporary cures as opposed to preventative strategies. Dapagliflozin, a sodium-glucose cotransporter-2 inhibitor (SGLT2i), was compared to placebo in heart failure patients with preserved ejection fraction (HFpEF). Positive results were expected, as SGLT2 inhibition was previously shown effective in patients with type 2 diabetes, kidney disease, or reducing heart failure. Dapagliflozin met its primary outcome: a reduction in the composite score of worsening heart failure or cardiovascular death. However, just like the EMPEROR-Preserved trial with another SGLT2i empagliflozin, the positive effect of dapagliflozin was mainly driven by a reduction in heart failure hospitalizations with no effect on cardiovascular death. Given that heart failure hospitalizations represent only a low number of all hospitalizations in patients with HFpEF, this high-cost drug class may be of low value in treating difficult-to-manage HFpEF. As baseline therapies have improved over time, it is harder to demonstrate big gains with newer expensive drugs. It is becoming less reasonable to warrant the spending of precious healthcare funds, unless significant improvements to health are clearly proven. Hence, there is an urgent need for preventative strategies to lower the incidences of CVD before patients become symptomatic and require costly care.

*Prevention is better than cure* was a key theme at this year’s European Society of Cardiology’s (ESC) Congress. Population-based screening has been viewed as one method to detect subclinical CVD. The DANCAVAS randomized trial assessed the efficacy of a comprehensive cardiovascular screen versus standard care in > 45,000 Danish men. Investigators found that screening was associated with a significant 11% reduction in mortality in 65- to 69-year-olds. Over a 5-year follow-up, prescriptions of antiplatelet and lipid-lowering agents significantly increased in the intervention arm. No differences in the outcomes in men aged > 70 years were observed, thus implying that the target age for screening should be < 70 years old. The eBRAVE trial utilized conventional smartphones to double the detection of treatment-relevant atrial fibrillation in older adults versus traditional care. Digital screening with a certified app more than doubled the rate of atrial fibrillation detection in both the randomization and cross-over phases of this trial. Both studies confirm that screening identified CVD earlier than traditional methods and may lead to better treatment outcomes.

Artificial intelligence (AI) offers the potential to enable better CVD diagnosis and management. Research findings have been promising, although questions remain regarding limited clinical implementation to date. During a keynote ESC presentation, Professor Brian Ference (Cambridge University, UK) presented the novel CAUSAL AI algorithm. CAUSAL AI combined deep and machine learning to accurately estimate the lifetime risk of major cardiac events among assessed participants when embedded into the JBS3 risk score, a standard tool used to estimate CVD risk. This algorithm more precisely predicted the benefit of lowering low-density lipoprotein (LDL) cholesterol, systolic blood pressure (SBP), or both on atherosclerotic CVD events in multiple clinical cohorts. These benefits started at any age and extended for any duration, with further analyses validating these original findings. CAUSAL AI’s findings agree with evidence from previous trials and Mendelian randomization studies, which stipulate that lowering LDL and SBP levels reduces the risk of atherosclerotic CVD. This new algorithm can improve existing risk prediction scores, which currently do not account for the causal effect of LDL and SBP in humans. Neither have risk scores accurately estimated baseline cardiovascular risk or the benefit of lowering LDL or SBP in atherosclerotic CVD.

While CAUSAL AI must be peer-reviewed and subsequently investigated in real-world populations, algorithms that learn the effects of modifiable disease causes have the potential to benefit many in society. It opens the door to transformative changes in primary prevention. That is “providing the essential information needed to inform individual treatment decisions about the optimal timing, duration, and intensity of lowering LDL and SBP to optimally prevent cardiovascular events for each person,” predicted Prof. Ference in his presentation. Extrapolating such AI into personalized medicine programs promises to provide actionable information to guide clinical decision-making. These precision algorithms could be targeted to at-risk individuals in early and mid-adulthood to minimize their likelihood of chronic disease progression. In the near future, these groups might hypothetically be prescribed specific lifestyle changes via smartphone apps to reduce their risk profile and likelihood of developing CVD.

Knowledge of likely successful preventative actions is a powerful tool to prevent CVD. But how does one then translate this knowledge into actionable and efficacious strategies? Well-established knowledge should be applied to elicit meaningful benefits, such as clinicians prescribing exercise as medicine for patients. Measures known to assist healthy living must be promoted and not scrapped, such as the UK Soft Drinks Industry Levy, currently under governmental review. Moreover, encouraging innovations like screening and predictive algorithms should be funded and trialed to determine if they can benefit whole populations. Only then could societies imagine alleviating disease burden and healthcare costs.

